# Supplementary material for: Does the Infectious Status of Aphids Influence Their Preference Towards Healthy, Virus-Infected and Endophytically Colonized Plants?
Source: Insects. 2020 Jul 11;11(7):435. doi: 10.3390/insects11070435 (PMC7412421; doi:10.3390/insects11070435)
Supplement: Supplementary file 1 [file insects-11-00435-s001.pdf]

insects MDPI

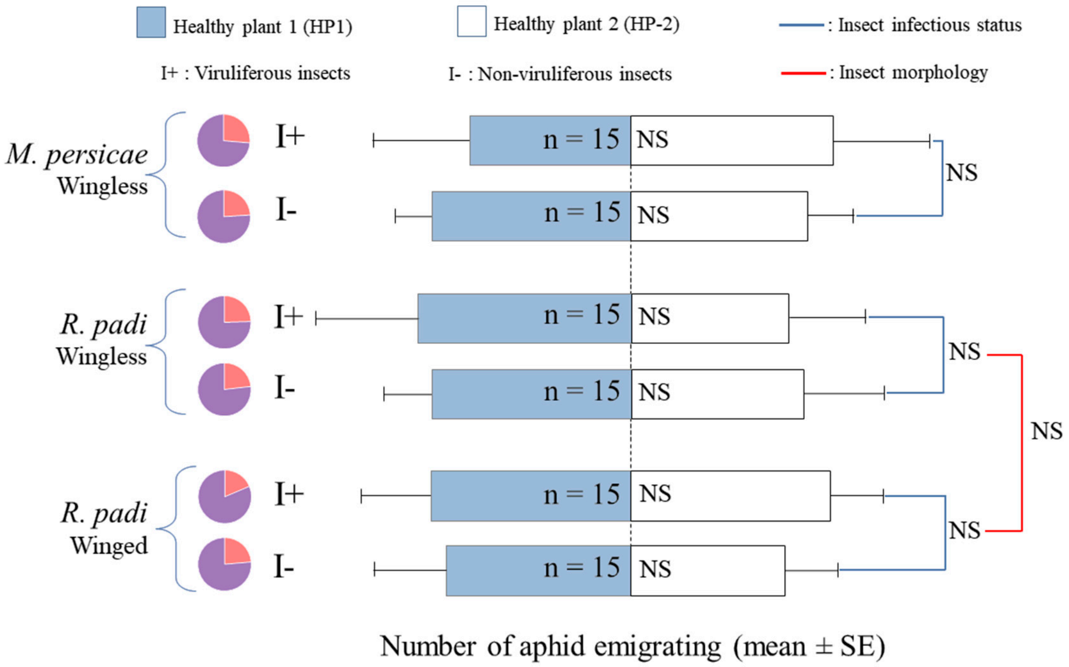

Figure 1. Preference tests (Mean ± SE) of insects between two healthy plants (HP). Each test was performed by releasing 20 viruliferous (I+) or non-viruliferous (I-) insects and observed after 60 min. Pie charts denote the proportion of responding (purple) versus non-responding individuals (orange). n: number of replicates; NS = not significant at $\alpha = 0.05$.

insects

MDPI

Table 1. Headspace volatile compounds of tobacco and wheat (mean ± SEM) in nanograms per g of fresh plant, sampled over 24 h from healthy plants (HP), virus-infected plants (i.e., tobacco infected by PLRV (VP-1) and wheat infected by BYDV (VP-2)) and endophytic entomopathogenic inoculated plants comprising *Metharizium acridum* inoculated plants (MP) and *Beauveria bassiana* inoculated plants (BP). Values with different letters within each plant model in the same raw are significantly different to each other ($p < 0.05$).

Compound	Chemical Family	Tobacco				Wheat			
		HP	VP-1	MP	BP	HP	VP-2	MP	BP
Glycerol.triacetate	Acetate	-	-	-	-	0 a	0 a	0.15 ± 0.06 b	0 a
3-Isopropenyl-2-methylcyclohexyl-acetate	Acetate	0 a	0.72 ± 0.30 b	0 a	0 a	-	-	-	-
2-Ethyl-3-hydroxyhexyl.2-methylpropanoate	Acid	-	-	-	-	0 a	0 a	0.14 ± 0.06 b	0 a
Bis(1-chloro-2-propyl)(3-chloro-1-propyl)phosphate	Acid	-	-	-	-	0 a	0 a	0.63 ± 0.26 b	0 a
1-Hexanol,-2-ethyl	Alcohol	-	-	-	-	2.53 ± 0.65 a	1.96 ± 0.62 a	2.87 ± 0.77 a	1.77 ± 0.57 a
Tridecan-1-ol	Alcohol	0 a	1.23 ± 0.52 b	0.65 ± 0.18 b	0 a	-	-	-	-
2-Propanol-1-chloro-phosphate-(3:1)	Alcohol	-	-	-	-	0 a	0 a	0.16 ± 0.07 b	0 a
Hexadecan-1-ol	Alcohol	0 a	1.25 ± 0.58 ab	1.69 ± 0.52 b	0 a	0 a	0 a	0.13 ± 0.06 b	0 a
Thunbergol	Alcohol	0 a	1.10 ± 0.31 b	0.40 ± 0.18 ab	0 a	-	-	-	-
Heptanal	Aldehyde	0 a	0 a	0.56 ± 0.17 b	1.37 ± 0.67 b	0 a	0 a	0 a	0.17 ± 0.07 b
Octanal	Aldehyde	0 a	0 a	0.35 ± 0.15 b	0 a	0 a	0 a	0.28 ± 0.07 b	0.31 ± 0.10 b
Nonanal	Aldehyde	0 a	0 a	7.53 ± 0.88 b	13.40 ± 2.61 c	0.83 ± 0.08 a	1.46 ± 0.31 ab	2.50 ± 0.09 b	10.26 ± 0.67 c
Decanal	Aldehyde	-	-	-	-	1.42 ± 0.23 a	1.92 ± 0.17 ab	2.42 ± 0.16 b	1.36 ± 0.15 a
Pentadecanal	Aldehyde	0 a	0 a	0.36 ± 0.16 b	0 a	-	-	-	-
Octadecanal	Aldehyde	12.35 ± 5.40 a	1.09 ± 0.46 ab	0 b	0 b	-	-	-	-
Formamide,-N,N-dibutyl	Amine	-	-	-	-	0 a	0.83 ± 0.22 b	0.59 ± 0.26 ab	0 a
4,8,13-Duvatriene-1,3-diol	Diol	0 a	1.90 ± 0.84 b	0 a	0 a	-	-	-	-
Ethylether	Ether	10.29 ± 4.64 a	0 b	0 b	4.31 ± 0.67 a	-	-	-	-
1-Pentene,-2-methyl	Hydrocarbon	0 a	0 a	0 a	9.34 ± 4.74 b	-	-	-	-
Benzene	Hydrocarbon	0 a	1.04 ± 0.59 b	0 a	0 a	-	-	-	-
Hexadecane	Hydrocarbon	0 a	1.10 ± 0.46 b	0 a	0 a	1.43 ± 0.18 a	1.49 ± 0.19 a	5.30 ± 0.74 b	1.33 ± 0.20 a
Heptadecane	Hydrocarbon	0 a	0.95 ± 0.67 b	0 a	0 a	-	-	-	-
Nonadecane	Hydrocarbon	-	-	-	-	0 a	0 a	0 a	0.18 ± 0.08 b
Eicosane	Hydrocarbon	0 a	1.37 ± 0.36 b	0 a	0 a	-	-	-	-
Solanone	Ketone	0 a	0 a	2.26 ± 0.53 b	5.02 ± 2.26 b	-	-	-	-
beta.-Ionone	Ketone	-	-	-	-	0 a	0 a	0.54 ± 0.14 b	0.73 ± 0.34 b
Dihydroactinidolide	Ketone	-	-	-	-	0 a	0 a	0 a	0.63 ± 0.18 b
6,10,14-Trimethylpentadecan-2-one	Ketone	-	-	-	-	1.31 ± 0.25 a	0.41 ± 0.17 a	0.37 ± 0.12 a	1.30 ± 0.54 a
Solavetivone	Ketone	0 a	0 a	0 a	1.17 ± 0.53 b	-	-	-	-
2-Pentadecanone,-6,10,14-trimethyl	Ketone	0 a	0.28 ± 0.12 b	0 a	0 a	-	-	-	-
Butylhydroxytoluene	Phenol	-	-	-	-	1.11 ± 0.46 a	0 b	0.20 ± 0.09 b	0 b
Alpha-Pinene	Terpene	-	-	-	-	0.77 ± 0.36 a	0 b	0 b	0.24 ± 0.11 ab
Neophytadiene	Terpene	12.64 ± 5.31 a	3.95 ± 1.12 a	3.81 ± 0.64 a	2.12 ± 0.65 a	-	-	-	-
Cembrene	Terpene	0 a	0.48 ± 0.22 b	0.57 ± 0.07 b	0 a	-	-	-	-
Unidentified-compound	-	0 a	0.18 ± 0.08 b	0 a	0 a	-	-	-	-

Table 2. Summary of pairwise comparisons of volatile profiles between different treatments for tobacco and wheat models. HP: healthy plants, VP: virus-infected plants (comprising tobacco infected by PLRV (VP-1) and wheat infected by BYDV (VP-2)), MP: *Metharizium acridum* inoculated plants (MP) and BP: *Beauveria bassiana* inoculated plants. "*" and "ns" for p≤0.05 and not significant at α = 0.05, respectively.

	**Tobacco**			**Wheat**		
	**F**	**p-adjusted**		**F**	**p-adjusted**	
HP vs VP	4.75	0.036	*	3.86	0.108	ns
HP vs MP	10.87	0.048	*	11.26	0.078	ns
HP vs BP	5.88	0.042	*	17.84	0.030	*
VP vs MP	7.56	0.060	ns	7.67	0.030	*
VP vs BP	8.03	0.048	*	19.99	0.036	*
BP vs MP	9.11	0.072	ns	22.90	0.030	*
